# The STRYDE limb lengthening nail is susceptible to mechanically assisted crevice corrosion: an analysis of 23 retrieved implants

**DOI:** 10.1080/17453674.2021.1927506

**Published:** 2021-06-08

**Authors:** Morten Stendahl Jellesen, Trine Nybo Lomholt, Rikke Quist Hansen, Troels Mathiesen, Carsten Gundlach, Søren Kold, Tobias Nygaard, Mindaugas Mikuzis, Ulrik Kähler Olesen, Jan Duedal Rölfing

**Affiliations:** a Department of Mechanical Engineering, Technical University of Denmark , Lyngby ;; b Materials and Product Testing, FORCE Technology , Brøndby ;; c Department of Physics, Technical University of Denmark , Lyngby ;; d Department of Orthopaedics, Interdisciplinary Orthopaedics, Aalborg University Hospital , Aalborg ;; e Department of Orthopaedics, Limb Lengthening and Bone Reconstruction Unit, Rigshospitalet , Copenhagen ;; f Orthopaedic Reconstruction and Children’s Orthopaedics, Aarhus University Hospital , Aarhus , Denmark

## Abstract

Background and purpose — We noted several adverse events in patients in whom the first version of the STRYDE limb-lengthening nail (NuVasive Specialized Orthopaedics, San Diego, CA) had been implanted. Pain, osteolysis, periosteal reactions, and cortical hypertrophy at the nail junction were noted. Here, we present the analysis of 23 retrieved STRYDE implants.

Materials and methods — We undertook visual inspection of the retrieved nails and screws, mechanical evaluation of the junction, micro-CT analyses, microscopic inspection of the bushing, screws, screw holes, and separated parts of the implants. Positive material identification (PMI) and energy-dispersive X-ray spectroscopy (EDS) were used to analyze the chemical composition. The hardness of the material was also investigated.

Results — 20/23 retrieved nails had visible signs of corrosion, i.e., discoloration at the telescopic junction. Micro-CT verified corrosion attacks in 12/12 scanned bushings. Corrosion, predominantly mechanically assisted crevice corrosion, was observed at the locking screws and screw holes in 20/23 nails. Biological material inside the nail was observed in addition to oozing from the junction of 2 nails during hardware removal, which was experimentally reproducible. Notably, the mechanical construction of the bushing changed from PRECICE P2 to STRYDE nails.

Interpretation — STRYDE nails are not hermetically sealed, and liquid can pass the bushing. Biodur 108 itself is corrosion resistant; however, mechanically assisted crevice corrosion of the bushing, locking screws, and screw holes may be aggravated due to manufacturing aiming for increased strength and hardness of the alloy.

Observing several adverse events, we recently published a nationwide cross-sectional analysis of all 30 STRYDE limb- lengthening nails (NuVasive, Specialized Orthopedics, San Diego, CA) that were implanted in Denmark (Rölfing et al. 2021a). 27/30 STRYDE nails have now been removed and we present data from metallurgical analysis of 23 of the retrieved implants.

## Materials and methods

We performed an analysis of all STRYDE nails, removed either routinely, due to complications, or preemptively due to our recent discovery of adverse events with this implant (Rölfing et al. 2021a). STRYDE nails from the 4 centers—Aarhus University Hospital, Aalborg University Hospital, Odense University Hospital, and Rigshospitalet, Copenhagen, Denmark—were visually inspected and photo documented (Figure 1, see Supplementary data). Based on these initial observations, the engineers (MSJ, TNL, RQH, TM) decided together with JDR which analyses should be performed for the individual nails. Representative samples of “worst” and “best” cases in terms of discoloration at the telescoping nail junction were further analyzed by either FORCE Technology (FT) and/or the Technical University of Denmark (DTU).

### Visual inspection and mechanical testing

All nails were inspected and photo documented by the surgeons, and after FT and DTU received these. Handling of the implants was not uniform; some implants were wiped with an ethanol cloth only; others were also washed in a surgical instrument dishwasher before photo documentation. Movement of the telescoping nail parts was assessed with manual compression and distraction of the nail (Video at https://youtu.be/CZiqmwgI_tI).

### Microscopic inspection

Microscopic inspection for signs of uniform and local corrosion was performed with a VHX-S650E digital microscope (Keyence Corporation, Osaka, Japan) with 20–100× magnification at DTU and a Leica MC 120 HD camera at FT. Microstructure examination was performed on cut sections of selected nails, followed by mounting in resin and subsequent grinding and polishing down to 1 µm diamond polishing. After etching in Vogels Sparbeize etchant, the microstructure was examined using a Leica DMI 5000 M metallographic microscope.

### Micro-CT

14 nails (12 STRYDE and 2 PRECICE P2.2 nails) were micro-CT scanned with a Nikon XT H 225 (Nikon Metrology Inc, Brighton, MI, USA; 160 kV, 125 µA, 1.0 mm copper filter, 801 views, 0.5 s exposure). Overview images of entire nails were recorded at 119.7 µm pixel size, while detailed scans were reconstructed at 19 or 23 µm isotropic resolution at the bushing for 12 nails, the O-rings for 5 nails and the ball bearing for 4 nails (Figures 2 and 3, see Supplementary data).

### Mechanical sectioning

5 STRYDE nails were mechanically sectioned at either FT or DTU.

### Hardness analysis

Hardness measurements were performed according to Vickers hardness test method described in DS/EN ISO 6507-1:2018 using a Struers Durascan 80A with a load of 2 kg (HV2).

### Positive materials identification (PMI)

The element weight percentage (wt.%) of the different components of the nail was assessed with X-ray fluorescence using a X-MET 8000 Optimum (Oxford Instruments, Abingdon, UK).

### Scanning electron microscopy (SEM)

SEM was performed using a ZEISS EVO MIK-015 BRUKER X-Flash DET—011 with 15 kV (Karl Zeiss Microscopy Deutschland GmbH, Oberkochen, Germany). Suspected biological material was evaluated with energy-dispersive X-ray spectroscopy (EDS) to verify high-levels of oxygen.

Data are presented as median (range).

### Ethics, funding, and conflicts of interest

The study was conducted with consent of the patients according to the Declaration of Helsinki and was approved by the local institutional review boards. The work conducted by FORCE Technology was financially supported by the Danish Agency for Higher Education and Science, Denmark. No other external financing was received. The authors declare no conflicts of interest.

## Results

30 STRYDE limb-lengthening nails (19 femoral and 11 tibial nails) were eligible for the study. The median lengthening of 30 bone segments of 27 patients, median age 20 (11–65) years was 35 (15–80) mm. For further details, please see Rölfing et al. (2021a).

3 nails are still implanted. Median time from implantation to hardware removal of the 27 retrieved nails was 12 (3.5–20) months. 23/27 nails were available for analysis, because 1 implant was lost to sonication and 3 nails were removed and discarded before we became aware of the observed adverse events. 1 of the 23 nails was returned to the manufacturer for further analysis after visual inspection and photo documentation had been performed.

**Figure 4.**
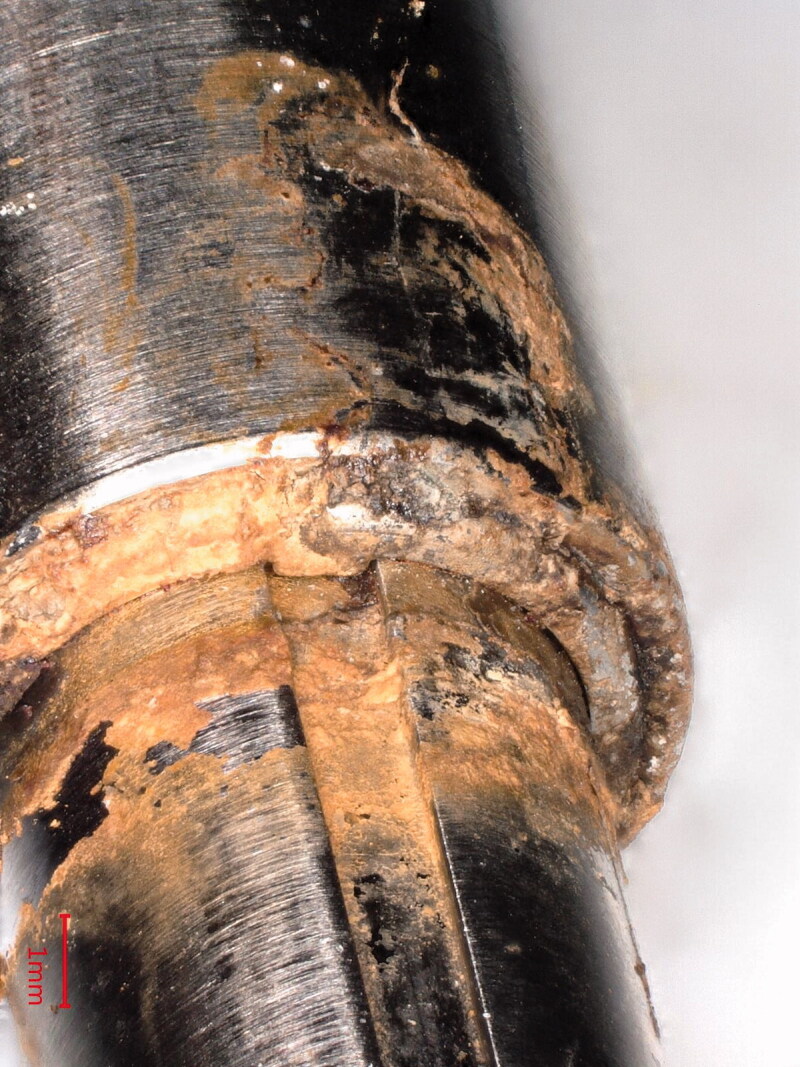
Digital microscopy of the junctional discoloration, i.e., corrosion products.

2 surgeons observed “oozing” of a brown substance from the telescopic nail junction during hardware removal in addition to the observation of junctional discoloration (Figure 4).

Moreover, 4 of the 27 retrieved STRYDE nails fractured, 3 before and 1 during hardware removal. All 4 patients were within the weight limit of the applied nail (Robbins and Paley 2020, Rölfing et al. 2021a, 2021b). Notably, the bone regenerate was deemed to be sufficiently healed in 1 patient, while 3 regenerates were insufficient. 2 PRECICE titanium nails (P2.2) were also analyzed to compare these with the STRYDE nails.

### Macroscopic inspection and photo documentation

Macroscopic inspection and photo documentation revealed discoloration at the telescoping junction in 20/23 implants (Figure 4) and 20/23 had corrosion at the locking screw holes (Table 1). Corrosion near the telescopic junction was seen as corrosion products leaking from the nail. The distraction rod itself did not show signs of uniform or pitting corrosion when inspected with light optical microscopy at 200x magnification. Attacks on the rod within the bushing were noted. Likewise empty screw holes were unaffected, whereas most screw holes in contact with screws suffered from material degradation. Degradation happened predominantly inside and on the load-bearing side of the hole with a smooth and hemispherical corrosion morphology. Screws thus showed indications of mechanically assisted crevice corrosion primarily on one side of the screw, but degradation on both sides was also observed (Figure 1, see Supplementary data).

**Table 1.** Symptoms, radiographic changes, and metallurgical characteristics of the 30 STRYDE-lengthened bone segments

Symptoms	
Late onset of pain^a^	8/30
Swelling^a^	3/30
Radiographic changes	
Junctional osteolysis^a^	19/30
Periosteal reaction^a^	12/30
Cortical hypertrophy^a^	12/30
Blood samples	
Elevated Cr blood levels^b^	1/15
Metallurgical features	
Naked eye visible junctional corrosion	20/23
Micro-CT verified junctional corrosion	12/12
Corrosion of screw hole/screws	20/23

Not all nails were available for all analyses, therefore n/N varies.

a Data previously reported (Rцlfing et al. 2021a).

b Chromium (Cr) levels at the time of nail removal. The wt.% of Cr and Mn are approximately the same in Biodur 108. However, Mn (and Mo) blood samples are not readily available in Denmark.

### Manual compression and distraction

Manual compression and distraction of the devices showed that 13/20 nails could be telescoped several millimeters with little force (Figure 5, see Supplementary data).

### Micro-CT

**Figure 6.**
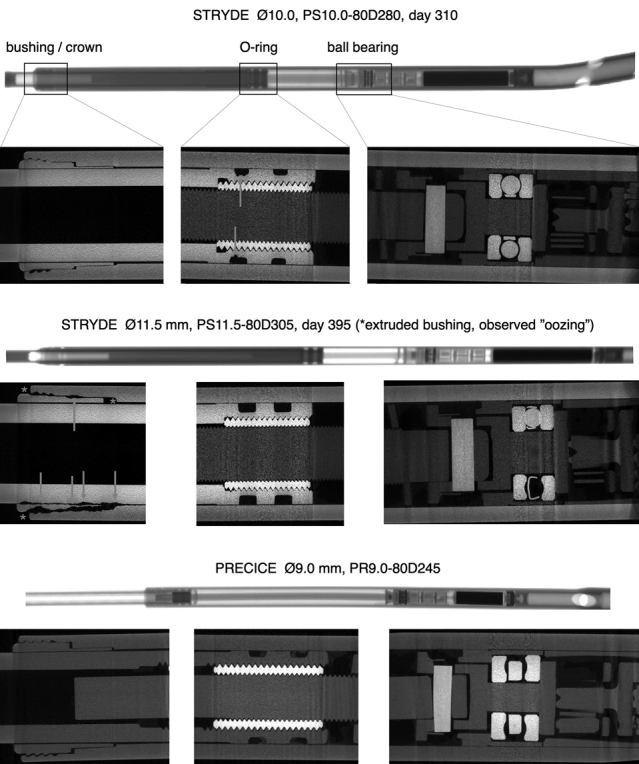
Micro-CT of 2 STRYDE and 1 PRECICE P2. Overview and detailed imaging of the bushing/crown, O-rings and ball bearing. Corrosion is seen around the bushing/crown in 12/12 nails scanned at this location (middle panel), corrosion at the O-ring was observed in 1/5 nails scanned at this location (top panel). Notably, no signs of corrosion were seen around the ball bearing in 4/4 scanned nails at this location. No corrosion was seen in the reference PRECICE P2 nail at any location. The mechanical components inside the housing appeared to be similar between STRYDE and PRECICE P2.

Micro-CT of 12 STRYDE nails documented corrosion attacks at the telescopic junction, primarily at the bushing, but also at the distraction rod and internal components of the nail surrounding the bushing (Figure 6).

Comparison of the scanned STRYDE and 2 scanned PRECICE P2.2 nails showed a similar mechanical construction; only the bushing seemed to be constructed differently (Figure 6). Close 360° contact between the housing and bushing were observed in PRECICE P2, while none of the 12 STRYDE nails had that feature (Figure 6, lower vs. top panel).

### Mechanical sectioning

**Figure 8.**
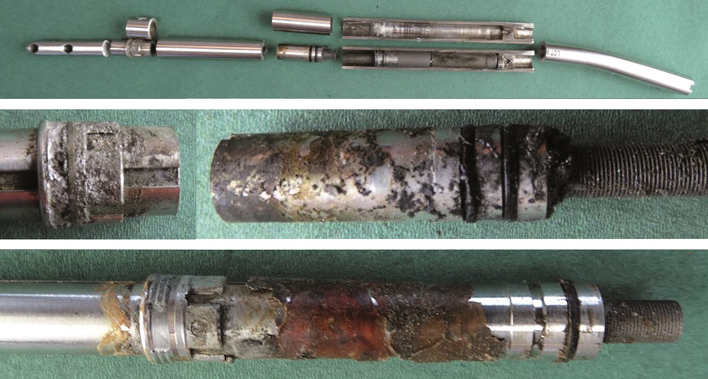
Examples of a sectioned STRYDE (top panel), corroded bushing (middle panel), and presence of biological material on internal components. Biological origin was verified with EDS analysis showing a high oxygen content (bottom panel).

Mechanical sectioning of 5 STRYDE nails, which were selected based on either visual inspection or micro-CT findings, i.e., worst and best cases, showed an internal titanium actuator pin with signs of dry lubricant of a crystalline appearance (Figure 7, see Supplementary data). EDS analysis of the crystalloid structure showed high contents of fluorine 35 wt.% and aluminum 24 wt.%. The presence of biologic material within the nail was confirmed by visual inspection and a high oxygen content determined by EDS analysis (Figure 8).

The O-ring sealing within the internal compartments of the nail appeared intact, besides 1 nail suffering from corrosion attack, also at this location. Interestingly, the bushing of that nail was not severely attacked by corrosion.

### Microscopic inspection

**Figure 9.**
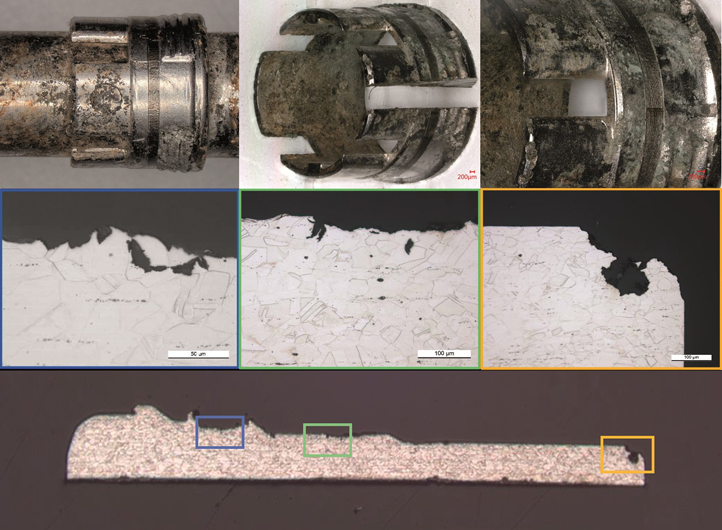
Corrosion attack at the bushing/crown. Digital microscopy (upper panels) and microscopy of the microstructure (middle and lower panels).

**Figure 10.**
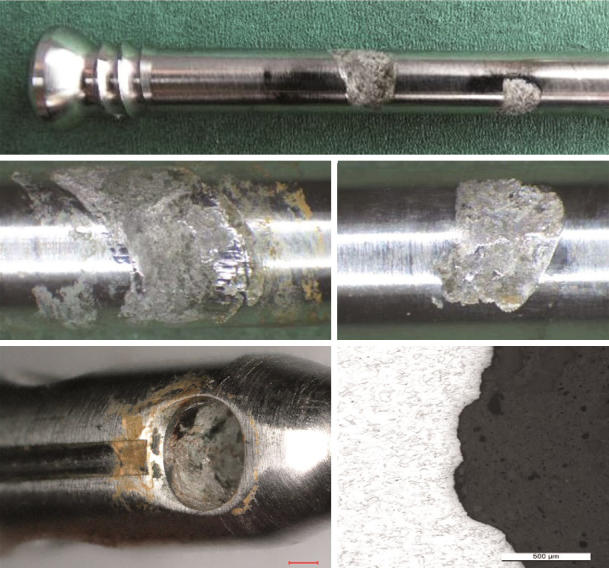
Mechanically assisted crevice corrosion/fretting and crevice corrosion at a locking screw, screw hole including microscopic image of the corrosion-attacked microstructure. For SEM imaging see Figures 12 and 13 in Supplementary data.

Microscopic inspection verified the mechanical damage and local corrosion of the microstructure both at the bushing (Figure 9) and at the screw and screw holes (Figure 10).

### PMI

PMI showed that the housing body, bushing, and locking screws fulfilled the Biodur 108 alloy ASTM 2229 specifications (ASTM 2021), i.e., content of manganese (Mn), chromium (Cr), molybdenum (Mo), and iron (Fe) (Table 2, see Supplementary data). Moreover, the visible discoloration, i.e., corrosion products, contained the same elements.

### Hardness analysis

Hardness analysis of the bushing, distraction rod, housing, and screws showed a hardness in the 410–445 HV range. This means that the manufactured nail has increased mechanical properties compared with the annealed conditions of the alloy (Figure 11, see Supplementary data).

### Distraction—retraction—distraction

Distraction—retraction—distraction of 2 nails using the fast distractor showed that the nails were not hermetically sealed. Air was pressed out of the nail, forming bubbles during distraction underwater. After subsequent retraction underwater and drying of the nail, the nail was distracted, which led to brown liquid leaking out of the telescoping junction (Figure 5, see Supplementary data).

## Discussion

The main findings of our study were:

1. 20/23 nails had visible signs of corrosion, i.e., discoloration at the telescopic junction. Micro-CT verified corrosion attacks in 12/12 scanned bushings.
2. Mechanically assisted crevice corrosion was also observed at the locking screws and screw holes in 20/23 nails.
3. STRYDE nails are not hermetically sealed. We found biological material and corrosion inside the nail and observed oozing from the junction of 2 nails during hardware removal, and this observation was experimentally reproducible.

In agreement with our results, Vogt et al. (2021) and Iliadis et al. (2021a) also reported corrosion after metallurgical analysis of retrieved STRYDE nails. While reports of implant failures in limb-lengthening nails are rare (Frost et al. 2021), the related magnetically controlled spinal growing rod devices (MAGEC, NuVasive, San Diego, CA, USA) have been highly investigated in recent years (Tang et al. 2019, Akbarnia and Mundis 2019, Agarwal et al. 2020, Joyce et al. 2020, Rushton et al. 2020, Tsirikos and Roberts 2020, Wei et al. 2020, Rushton et al. 2021). In these spinal implants, wear debris causing metallosis, i.e., discoloration of the surrounding soft tissue, originally drew surgeons’ attention to the matter. Later, after extensive analyses, increased blood levels of metal ions and breakage of internal components as well as corrosion were reported. Nonetheless, the lengthening process with MAGEC growing rods is almost pain-free and may be a gentler treatment option with high patient satisfaction compared with sequential open spinal lengthening procedures (Skov et al. 2019, 2020a). Many surgeons therefore still advocate the use of MAGEC implants, despite the call for evaluation of the cost-effectiveness and safety of these devices (Rushton et al. 2020, Skov et al. 2020b, Tsirikos and Roberts 2020).

In contrast to spinal growing rods, limb-lengthening nails are not surrounded by soft tissue, but surrounded by bone. Our current working hypothesis regarding STRYDE is that internal and junctional corrosion and its products cause a toxic environment leading to osteolysis. In an effort to protect its mechanical integrity, the bone forms periosteal reaction (predominantly onion-skin layered) before cortical hypertrophy occurs, which is likely the end result—unless the cortical destruction is substantial (Rölfing et al 2021a).

Whereas MAGEC and PRECICE are made of a titanium alloy, STRYDE is made of stainless steel Biodur 108. In general, austenitic stainless steel (as in the AISI 300 series) is known to offer appropriate corrosion resistance for many applications including orthopedic implants. The resistance to uniform corrosion is explained by the existence of a dense chromium oxide forming a passive film on the surface. Nevertheless, the passive film can locally degrade, e.g., by small-amplitude wearing movements or due to the formation of a local aggressive environment in occluded regions prone to depletion of oxygen (crevice corrosion). High nitrogen containing austenitic stainless steel (HNS) such as Biodur 108 maintains the austenitic structure by the addition of nitrogen and manganese without having a major nickel content. Having nitrogen dissolved in the austenitic stainless steel results in higher strength compared with nickel containing austenitic stainless steel. HNS is also reported to offer improved pitting and crevice corrosion resistance in the solution-annealed state compared with the AISI 300 series (Lim et al. 2001, Baba and Katada 2006). The effect of cold-working (metalworking at ambient temperature) of HNS will further increase the strength and hardness of the alloy; however, cold-working has proven to have an unfavorable effect on pitting and crevice corrosion resistance (Kamachi Mudali et al. 2002, Wang et al. 2017). The effect on corrosion behavior of having an increased amount of manganese at the expense of nickel in the stainless-steel alloy is lower pitting and crevice corrosion resistance as well as a lower repassivation rate if the passive film is broken down (Lim et al. 2001, Toor et al. 2007). Other important factors affecting the corrosion resistance of HNS alloys are the metallurgical structure, a fine-grained austenitic structure that is free of ferrite, chi, and sigma phases, the inclusion content, and the risk of carbonitride formation (ASTM F2229).

A possible explanation for corrosion near the telescopic junction is that corrosion initiates at the interfaces of the bushing where small-amplitude movements can produce disruption of the passive film causing metal degradation and crevice formation. As the environment in the crevice becomes more aggressive it leads to further degradation of the bushing and other parts of the telescopic junction.

Mechanically assisted crevice corrosion may also explain the material degradation inside the screw holes. Weight-bearing may initiate this process by causing small-amplitude movements between the screw and screw hole, initiating fretting. Subsequently, a larger crevice is formed where the distraction rod inside the screw hole is attacked due to increased aggressivity of the environment inside the crevice between screw and screw hole. Moreover, galvanic corrosion could be ruled out, because the housing, bushing, and screw were made from the same metal, i.e., Biodur 108.

An analysis of retrieved PRECICE nails documented improvements from the first version to the current version (Panagiotopoulou et al. 2018). However, in line with our findings regarding STRYDE, these authors also noted biological material within PRECICE nails, while internal corrosion was confined to the early versions. They also compared their findings with MAGEC spinal growing rods, highlighting that there are fundamental differences including the planned removal of limb-lengthening nails after bony consolidation (Panagiotopoulou et al. 2018, Rushton et al. 2020). They therefore state that it is “unlikely that a corrosive process will have sufficient time to cause an actuator pin fracture or other internal mechanism damage that compromises the use of the implant.” This notion is partially supported by Eltayeby et al. (2021) reporting on 102 retrieved nails, whereof 57/65 PRECICE P2 and 29/37 PRECICE P1 were still functioning after a median implantation time of 15 (4–47) months prior to hardware removal and testing. Albeit not the primary outcome of that study, the authors did not mention any signs of corrosion and only excluded 1 broken nail. Neither do they mention significant damage, or corrosion of the tested nails. Interestingly, Lee et al. (2017) describe broken bushings of PRECICE nails, but no corrosion. However, based on these studies and the body of evidence, clinical application of the titanium PRECICE nail is relatively safe (Alrabai et al. 2017, Calder et al. 2019, Horn et al. 2019, Hammouda et al. 2020, Morrison et al. 2020, Nasto et al. 2020, Frost et al. 2021, Iliadis et al 2021b).

To our knowledge, we have reported 4 out of 5 broken STRYDE nails in the literature (Johnson et al. 2021, Rölfing et al. 2021a, 2021b). The present study included analysis of our 4 broken nails, but we were unable to determine the cause of breakage through locking screw holes (n = 3) and where the magnet resides (n = 1).

Limitations of our study include that we chose which nail underwent which analyses based on an initial macroscopic inspection. Ideally, all nails should have undergone a standardized sequence of all analyses. Furthermore, handling of the implants immediately after removal was not standardized at the different centers, i.e., cleansing with saline water and/or ethanol wipes or cleaning in a surgical dishwasher. Nonetheless, all available nails underwent visual inspection and 12 STRYDE and 2 PRECICE P2.2 nails were analyzed with micro-CT. The external validity of our study is therefore more substantial than a previous micro-CT investigation of 2 P1, 1 P2.0, and 1 P2.1 PRECICE nails (Panagiotopoulou et al. 2018).

Another limitation is that we did not systematically evaluate whether the retrieved nails were still functioning (Eltayeby et al. 2021). The major strength of our study is that we can document correlation of the metallurgical analyses with the previously reported clinical and radiographic findings (Rölfing et al. 2021a).

In conclusion, we report corrosion causing internal and external damage to the nail, extrusion of the bushing, biological material within the nail, and the fact that fluid is able to pass the corroded bushing. The latter observations underline that the STRYDE nail is not hermetically sealed. Finally, the findings of our present study correlate with the radiographic changes and clinical symptoms noted in our previous study within the same cohort (Rölfing et al. 2021a). While our observational and experimental studies may not be able to determine a causal relation between corrosion leading to clinical symptoms and radiological findings, the covariation and temporal precedence make causality likely.

## Supplementary data

Table 2 and Figures 1–3, 5, 7 and 11–13 are available as supplementary data in the online version of this article, http://dx.doi.org/10.1080/17453674.2021.1927506

## References

[CIT0001] Agarwal A, Kelkar A, Agarwal A G, Jayaswal D, Jayaswal A, Shendge V. Device-related complications associated with MAGEC rod usage for distraction-based correction of scoliosis. Spine Surg Relat Res 2020; 4(2): 148–51. 32405561 10.22603/ssrr.2019-0041 PMC7217671

[CIT0002] Akbarnia B A, Mundis G M. Magnetically controlled growing rods in early onset scoliosis. Orthopade 2019; 48(6): 477–85. 31172228 10.1007/s00132-019-03755-0

[CIT0003] Alrabai H M, Gesheff M G, Conway J D. Use of internal lengthening nails in post-traumatic sequelae. Int Orthop 2017; 41(9): 1915–23. 28389837 10.1007/s00264-017-3466-6

[CIT0004] ASTM F2229-21, Standard Specification for Wrought, Nitrogen Strengthened 23Manganese-21Chromium-1Molybdenum Low-Nickel Stainless Steel Alloy Bar and Wire for Surgical Implants (UNS S29108). West Conshohocken, PA: ASTM International; 2021.

[CIT0005] Baba H, Katada Y. Effect of nitrogen on crevice corrosion in austenitic stainless steel. Corros Sci 2006; 48(9): 2510–24.

[CIT0006] Calder P R, McKay J E, Timms A J, Roskrow T, Fugazzotto S, Edel P, Goodier W D. Femoral lengthening using the Precice intramedullary limb-lengthening system: outcome comparison following antegrade and retrograde nails. Bone Joint J 2019; 101-B(9): 1168–76. 31474141 10.1302/0301-620X.101B9.BJJ-2018-1271.R1

[CIT0007] Eltayeby H H, Alrabai H M, Jauregui J J, Shabtai L Y, Herzenberg J E. Post-retrieval functionality testing of PRECICE lengthening nails: the “sleeper” nail concept. J Clin Orthop Trauma 2021; 14: 151–5. 33717907 10.1016/j.jcot.2020.06.005 PMC7920018

[CIT0008] Frost M, Rahbek O, Traerup J, Ceccotti A A, Kold S. Systematic review of complications with externally controlled motorized intramedullary bone lengthening nails (FITBONE and PRECICE) in 983 segments. Acta Orthop 2021; 91(1): 120–7. 10.1080/17453674.2020.1835321 PMC7919879 33106069

[CIT0009] Hammouda A I, Szymczuk V L, Gesheff M G, Mohamed N S, Conway J D, Standard S C, McClure P K, Herzenberg J E. Acute deformity correction and lengthening using the PRECICE magnetic intramedullary lengthening nail. J Limb Lengthen Reconstr 2020; 6: 20–7.

[CIT0010] Horn J, Hvid I, Huhnstock S, Breen A B, Steen H. Limb lengthening and deformity correction with externally controlled motorized intramedullary nails: evaluation of 50 consecutive lengthenings. Acta Orthop 2019; 90(1): 81–7. 30371122 10.1080/17453674.2018.1534321 PMC6366464

[CIT0011] Iliadis A D, Wright J, Stoddart M T, Goodier W D, Calder P. Early results from a single centre’s experience with the STRYDE nail. Bone Joint J. 2021a. In press (personal communication) 10.1302/0301-620X.103B6.BJJ-2020-2165.R1 34058877

[CIT0012] Iliadis A D, Palloni V, Wright J, Goodier D, Calder P. Pediatric lower limb lengthening using the PRECICE nail: our experience with 50 cases. J Pediatr Orthop 2021b; 41(1): e44-9. 32947442 10.1097/BPO.0000000000001672

[CIT0013] Johnson M A, Karkenny A J, Arkader A, Davidson R S. Dissociation of a femoral intramedullary magnetic lengthening nail during routine hardware removal. JBJS Case Connect 2021; 11(1): 1–5. 10.2106/JBJS.CC.20.00950 33684083

[CIT0014] Joyce T J, Smith S L, Kandemir G, Rushton P R P, Fender D, Bowey A J, Gibson M J. The NuVasive MAGEC rod urgent field safety notice concerning locking pin fracture: how does data from an independent explant center compare? Spine 2020; 45(13): 872–6. 32539289 10.1097/BRS.0000000000003439

[CIT0015] Kamachi Mudali U, Shankar P, Ningshen S, Dayal R K, Khatak H S, Raj B. On the pitting corrosion resistance of nitrogen alloyed cold worked austenitic stainless steels. Corros Sci 2002; 44(10): 2183–98.

[CIT0016] Lee D H, Kim S, Lee J W, Park H, Kim T Y, Kim H W. A comparison of the device-related complications of intramedullary lengthening nails using a new classification system. Biomed Res Int 2017; 2017: 8032510. 29130046 10.1155/2017/8032510 PMC5654310

[CIT0017] Lim Y S, Kim J S, Ahn S J, Kwon H S, Katada Y. The influences of microstructure and nitrogen alloying on pitting corrosion of type 316L and 20 wt.% Mn-substituted type 316L stainless steels. Corros Sci 2001; 43(1): 53–68.

[CIT0018] Morrison S G, Georgiadis A G, Huser A J, Dahl M T. Complications of limb lengthening with motorized intramedullary nails. J Am Acad Orthop Surg 2020; 28(18): e803-9. 32520902 10.5435/JAAOS-D-20-00064

[CIT0019] Nasto L A, Coppa V, Riganti S, Ruzzini L, Manfrini M, Campanacci L, Palmacci O, Boero S. Clinical results and complication rates of lower limb lengthening in paediatric patients using the PRECICE 2 intramedullary magnetic nail: a multicentre study. J Pediatr Orthop B 2020; 29(6): 611–17. 31904740 10.1097/BPB.0000000000000651

[CIT0020] Panagiotopoulou V C, Davda K, Hothi H S, Henckel J, Cerquiglini A, Goodier W D, Skinner J, Hart A, Calder P R. A retrieval analysis of the Precice intramedullary limb lengthening system. Bone Joint Res 2018; 7(7): 476–84. 30123497 10.1302/2046-3758.77.BJR-2017-0359.R1 PMC6076355

[CIT0021] Robbins C, Paley D. Stryde Weight-bearing Internal Lengthening Nail. Tech Orthop 2020; 35(3): 201–8.

[CIT0022] Rölfing J D, Kold S, Nygaard T, Mikuzis M, Brix M, Faergemann C, Gottliebsen M, Davidsen M, Petruskevicius J, Olesen U K. Pain, osteolysis, and periosteal reaction are associated with the STRYDE limb lengthening nail: a nationwide cross-sectional study. Acta Orthop 2021a; epub ahead of print. doi.org/10.1080/17453674.2021.1903278 PMC8428270 33757381

[CIT0023] Rölfing J D, Bünger M, Petruskevicius J, Abood A. Removal of broken Stryde limb lengthening nails. Orthop Traumatol Surg Res 2021b; in press. 10.1016/j.otsr.2021.102958 33965599

[CIT0024] Rushton P R P, Smith S L, Kandemir G, Forbes L, Fender D, Bowey A J, Gibson M J, Joyce T J. Spinal lengthening with magnetically controlled growing rods. Spine 2020; 45(3): 170–6. 31513114 10.1097/BRS.0000000000003215

[CIT0025] Rushton P R P, Smith S L, Fender D, Bowey A J, Gibson M J, Joyce T J. Metallosis is commonly associated with magnetically controlled growing rods: results from an independent multicentre explant database. Eur Spine J 2021; epub ahead of print. doi.org/10.1007/s00586-021-06750-2 33544222

[CIT0026] Skov S T, Bünger C, Rölfing J D, Hansen E S, Høy K, Valencius K, Hemig P, Li H. High global satisfaction in magnetically controlled elongations in 29 early-onset scoliosis patients versus primary spinal fusion in 20 adolescent idiopathic scoliosis patients. Open J Orthop Rheumatol 2019; 4(1): 005–9.

[CIT0027] Skov S T, Bünger C, Li H, Vigh-Larsen M, Rölfing J D. Lengthening of magnetically controlled growing rods caused minimal pain in 25 children: pain assessment with FPS-R, NRS, and r-FLACC. Spine Deform 2020a; 8(4): 763–70. 32170659 10.1007/s43390-020-00096-3

[CIT0028] Skov S T, Li H, Hansen E S, Høy K, Helmig P, Rölfing J D, Bünger C. New growth rod concept provides three dimensional correction, spinal growth, and preserved pulmonary function in early-onset scoliosis. Int Orthop 2020b; 44(9): 1773–83. 32494843 10.1007/s00264-020-04604-y

[CIT0029] Tang N, Zhao H, Shen J-X, Zhang J-G, Li S-G. Magnetically controlled growing rod for early-onset scoliosis: systematic review and meta-analysis. World Neurosurg 2019; 125: e593-601. 30716487 10.1016/j.wneu.2019.01.136

[CIT0030] Toor I-H, Park K J, Kwon H. Manganese effects on repassivation kinetics and SCC susceptibility of high Mn-N austenitic stainless steel alloys. J Electrochem Soc 2007; 154(9): C494.

[CIT0031] Tsirikos A I, Roberts S B. Magnetic controlled growth rods in the treatment of scoliosis: safety, efficacy and patient selection. Med Devices Evid Res 2020; 13: 75–85. 10.2147/MDER.S198176 PMC7085947 32256128

[CIT0032] Vogt B, Rödl R, Gosheger G, Schulze M, Hasselmann J, Fuest C, Toporowski G, Laufer A, Frommer A. Focal osteolysis and corrosion at the junction of Precice Stryde intramedullary lengthening device: preliminary clinical, radiographic and metallurgic analysis of 57 lengthened segments. Bone Joint Res 2021 (under review/personal communication). 10.1302/2046-3758.107.BJR-2021-0146.R1 PMC8333033 34269599

[CIT0033] Wang Q, Zhang B, Ren Y, Yang K. Eliminating detrimental effect of cold working on pitting corrosion resistance in high nitrogen austenitic stainless steels. Corros Sci 2017; 123: 351–5.

[CIT0034] Wei J Z, Hothi H S, Morganti H, Bergiers S, Dal Gal E, Likcani D, Henckel J, Hart A J. Mechanical wear analysis helps understand a mechanism of failure in retrieved magnetically controlled growing rods: a retrieval study. BMC Musculoskelet Disord 2020; 21(1): 519. 32758204 10.1186/s12891-020-03543-4 PMC7409688

